# Establishing *Salvia miltiorrhiza*-Derived Exosome-like Nanoparticles and Elucidating Their Role in Angiogenesis

**DOI:** 10.3390/molecules29071599

**Published:** 2024-04-03

**Authors:** Shuya Zhang, Jiaxuan Xia, Ying Zhu, Meichen Dong, Jianxin Wang

**Affiliations:** 1 Department of Pharmaceutics, School of Pharmacy, Fudan University & Key Laboratory of Smart Drug Delivery, Ministry of Education, MOE Innovative Center for New Drug Development of Immune Inflammatory Diseases, Fudan University, Shanghai 201203, China; 19211030018@fudan.edu.cn (S.Z.); xjxjade@163.com (J.X.); mcdong22@m.fudan.edu.cn (M.D.); 2 Department of Integrative Oncology, Fudan University Shanghai Cancer Center, Shanghai 200032, China; 21111230052@m.fudan.edu.cn

**Keywords:** Danshen-derived exosome-like nanoparticles, exosomes, angiogenesis promoters, myocardial ischemia–reperfusion injury

## Abstract

Exosomes are multifunctional, cell-derived nanoscale membrane vesicles. Exosomes derived from certain mammalian cells have been developed as angiogenesis promoters for the treatment of myocardial ischemia–reperfusion injury, as they possess the capability to enhance endothelial cell proliferation, migration, and angiogenesis. However, the low yield of exosomes derived from mammalian cells limits their clinical applications. Therefore, we chose to extract exosome-like nanoparticles from the traditional Chinese medicine *Salvia miltiorrhiza*, which has been shown to promote angiogenesis. *Salvia miltiorrhiza*-derived exosome-like nanoparticles offer advantages, such as being economical, easily obtainable, and high-yielding, and have an ideal particle size, Zeta potential, exosome-like morphology, and stability. *Salvia miltiorrhiza*-derived exosome-like nanoparticles can enhance the cell viability of Human Umbilical Vein Endothelial Cells and can promote cell migration and improve the neovascularization of the cardiac tissues of myocardial ischemia–reperfusion injury, indicating their potential as angiogenesis promoters for the treatment of myocardial ischemia–reperfusion injury.

## 1. Introduction

Myocardial ischemia–reperfusion (MI/R) injury is mainly attributed to the generation of a large amount of reactive oxygen species and the activation of inflammatory responses during the process of coronary artery reperfusion therapy for acute myocardial infarction [1,2]. This leads to oxidative stress damage to myocardial tissues. MI/R injury can result in pathological processes, such as myocardial cell necrosis, inflammation, fibrosis, etc., severely affecting cardiac function and the quality of life of patients [3,4]. Reducing MI/R injury has become an urgent scientific challenge. Current therapeutic strategies for MI/R injury primarily include the use of antioxidants to alleviate oxidative stress damage [5,6], immunosuppressants to reduce inflammatory reactions [7], and stem cell transplantation techniques for myocardial repair [8,9]. However, the efficacy of drug therapy is limited and cannot reverse the progression of heart failure after myocardial infarction, and stem cell therapy also has safety risks [10,11]. Angiogenesis promoters can facilitate the formation of new blood vessels and enhance the repair of existing blood vessels, which can improve the pathological and physiological processes of MI/R injury and potentially promote the regeneration and repair of myocardial tissues [12,13]. These drugs can act through various pathways, including promoting endothelial cell proliferation and migration, stimulating the release of vascular growth factors, and regulating vascular generation-related signaling pathways. Therefore, the development of angiogenesis promoters for the treatment of MI/R injury is an effective strategy [14,15].

As research on MI/R injury deepens, scientists are focusing on the potential of exosomes in the treatment of MI/R injury [16]. Exosomes are membrane vesicles secreted by cells and have been shown to be effective carriers of biological signal transmissions involving myocardial function and mediate the local and distal communication of the heart under physiological and pathological conditions [17,18,19]. Exosomes can be released from various cells, including myocardial cells, cardiac fibroblasts, endothelial cells, inflammatory cells, and various stem cells, thereby participating in processes such as cardiac protection in the body [20]. On the other hand, exosomes can be secreted from the myocardium to the circulatory system, mediating the connection between the heart and other tissues and cells. For example, exosomes released from ischemic myocardial cells can be delivered to endothelial cells, enhancing their proliferation, migration, and angiogenesis. Exosomes derived from mammalian cells have significant effects on improving cardiac function, reducing myocardial fibrosis, and stimulating angiogenesis [21]. Studies have found that exosome-derived miRNAs can promote angiogenesis after myocardial ischemia in animals [22,23,24]. Inspired by this, researchers have used exosomes as angiogenic agents for MI/R injury treatment to repair defective cardiac tissues.

Although mammalian cell-derived exosomes have diverse functions and wide applications, the extraction of mammalian cell-derived exosomes usually requires long-term cell culture, and their clinical translation faces challenges, such as a low yield and difficulty in scaling up production. Secondly, the use of exosomes from allogeneic cells also poses certain immunogenicity issues. Moreover, mammalian cell-derived exosomes in the treatment of MI/R injury are usually administered by local injection, which greatly limits their clinical applications. Compared with mammalian cell culture media, extracting exosome-like nanoparticles from plants will greatly increase the yield [25,26]. It has been reported that exosome-like nanoparticles can be isolated from plants such as ginger, ginseng, and *Panax notoginseng*, which have similar structural characteristics to exosomes derived from mammalian cells and have been applied in the treatment of inflammation and cardiovascular and cerebrovascular diseases [27,28,29]. Plant-derived exosome-like nanoparticles may be an ideal substitute for exosomes. Compared to mammalian-derived exosomes, plant-derived exosome-like nanoparticles also have advantages, such as oral availability and the ability to target disease sites depending on the nanoscale size via an intravenous injection [30,31].

*Salvia miltiorrhiza*, a traditional Chinese medicine, promotes blood circulation and alleviates blood stasis. As an angiogenesis promoter, it has a long history of applications in the prevention and treatment of cardiovascular diseases [32,33]. Its monomeric compounds and compound formulations have been developed into tablets, injections, lyophilized powders, soft capsules, and pills, which are widely used in the treatment of cardiovascular and cerebrovascular diseases. Modern pharmacology and clinical studies have shown that *Salvia miltiorrhiza* has various functions in preventing and treating MI/R injury, such as promoting endothelial cell proliferation and microvascular regeneration, but its effect is mild, and it is mostly used as an adjuvant therapy [34,35].

Based on this, we hope to develop a novel plant-derived exosome-like nanoparticle—*Salvia miltiorrhiza*-derived exosome-like nanoparticles (DDNs)—with the hope to retain the pharmacological activity of *Salvia miltiorrhiza* while utilizing the characteristics of plant-derived exosome-like nanoparticles to promote angiogenesis and apply it to the treatment of MI/R injury. This research focuses on the isolation and characterization of DDNs and in vitro and in vivo angiogenic efficacy and in vivo safety studies of DDNs.

## 2. Results

### 2.1. Isolation and Purification of Salvia miltiorrhiza-Derived Exosome-like Nanoparticles

**Figure 1:**
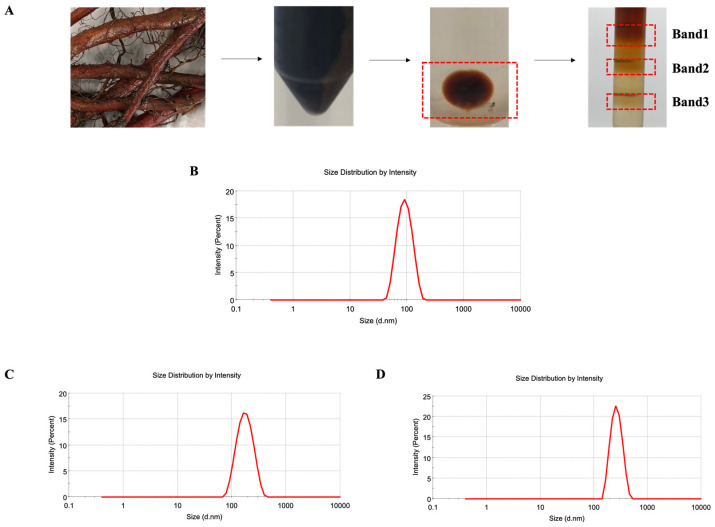
Isolation and purification of DDN. (**A**) Diagram of the extraction process; (**B**) size distribution of Band1, detected by DLS; (**C**) size distribution of Band2, detected by DLS; (**D**) size distribution of Band3, detected by DLS.

Based on the typical enrichment of exosomes in density bands ranging from 1.13 to 1.20 g/mL [36], a DDN is expected to accumulate in the layer of 8% and 30% sucrose solutions. As shown in Figure 1A, following ultracentrifugation, there was a clear precipitation of exosome-like nanoparticles at the bottom of the centrifuge tubes. Furthermore, after sucrose density gradient centrifugation purification, the exosome-like nanoparticles were noticeably enriched in the layer of 8% and 30% sucrose solutions. Typically, exosomes extracted using sucrose density gradient centrifugation have a particle size of less than 150 nm [37]. For further characterization, the solutions from the layers of 8% and 30%, 30% and 45%, and 45% and 60% sucrose were, respectively, collected and labeled as Band1, Band2, and Band3, and we measured the particle size using dynamic light scattering (DLS). The results, depicted in Figure 1B–D, show that the average particle size of Band1 was 83.61 nm with a PDI of 0.107, Band2 was 157.6 nm with a PDI of 0.141, and Band3 was 264.5 nm with a PDI of 0.238. Band1, enriched in the layer of the 8% and 30% sucrose solutions, exhibited the characteristic particle size of exosome-like nanoparticles with a uniform size distribution. Therefore, Band1 represents the successfully separated and purified DDN obtained through ultracentrifugation and sucrose density gradient centrifugation.

### 2.2. Characterization of Salvia miltiorrhiza-Derived Exosome-like Nanoparticles

**Figure 2:**
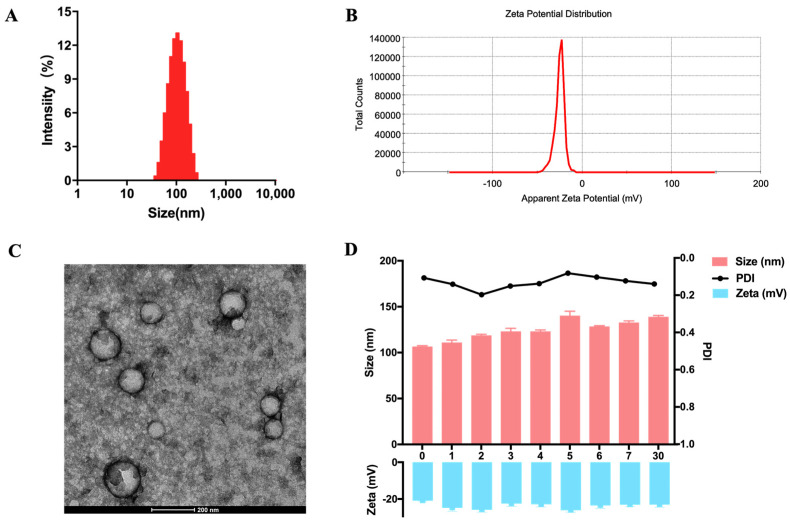
Characterization of DDN. (**A**) Size distribution of DDN, detected by DLS; (**B**) zeta potential of DDN, detected by DLS; (**C**) representative electron microscope image of DDN, scale bar = 200 nm; (**D**) stability of DDN (*n* = 3).

After freeze-drying, the DDN was stored in a −80 °C ultra-low temperature freezer and reconstituted in PBS before the subsequent experiments. The purified DDNs were characterized by DLS with an average particle size of 105.15 nm and low polydispersity (Figure 2A), and the Zeta potential of the DDNs was determined to be −25.2 mV (Figure 2B). The particle size of the DDN after freeze-drying and reconstitution was slightly increased compared to that of the freshly extracted DDN but still fell within the range of characteristic exosome sizes. The Zeta potential of the DDN was negative and similar to the cell membrane potential. A transmission electron microscopy (TEM) analysis revealed that a majority of the DDN, localized at the interface of 8% and 30% sucrose, exhibited a predominantly spherical shape with a membrane-enclosed vesicle-like structure (Figure 2C). DDN quantification was performed using protein concentration via a BCA protein assay and particle concentration by NTA. It was observed that the DDN was enriched in the extracts of *Salvia miltiorrhiza* roots (approximately 961 mg/kg of *Salvia miltiorrhiza* and 4.92 × 10^14^ particles/kg of *Salvia miltiorrhiza*), indicating a substantial production of the DDN obtained through ultracentrifugation and sucrose density gradient centrifugation. During a one-month storage period at −80 °C, the particle size of the DDN fluctuated between 100 nm and 140 nm, while the Zeta potential fluctuated between −20 mV and −25 mV, demonstrating good stability of DDNs under prolonged storage at −80 °C.

### 2.3. Cellular Uptake of Salvia miltiorrhiza-Derived Exosome-like Nanoparticles

**Figure 3:**
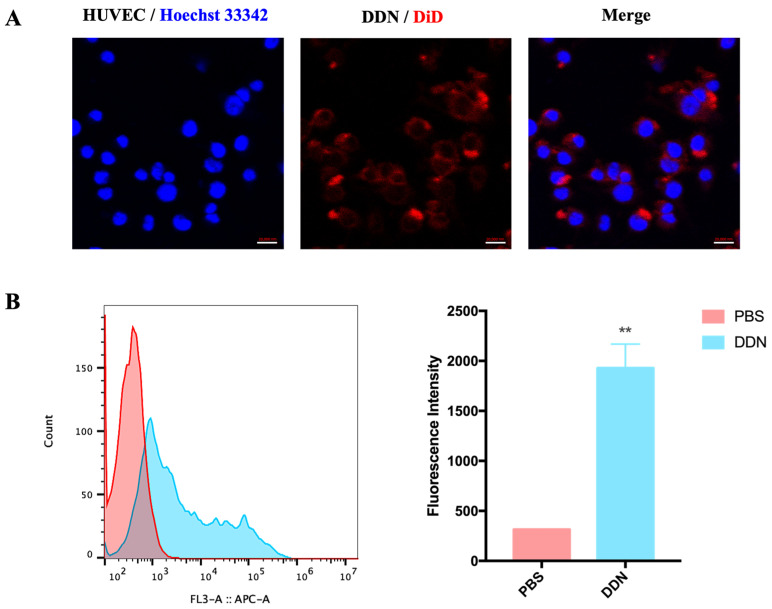
Cellular uptake of DDN by HUVECs. (**A**) Confocal image of HUVECs uptaking DDN, scale bar = 20 μm; (**B**) cellular uptake of DDN by HUVECs, detected by flow cytometry (*n* = 3). (** *p* < 0.01).

The cellular uptake of a DDN is a prerequisite for its therapeutic efficacy. Firstly, confocal laser scanning microscopy was employed to observe the uptake of the DDN by Human Umbilical Vein Endothelial Cells (HUVECs). The results in Figure 3A demonstrate that a DiD-DDN can be taken up by HUVECs, with the cellular localization of the DiD-DDN observed in the cytoplasm. Subsequently, flow cytometry was used to assess the uptake of the DiD-DDN by the HUVECs. As shown in Figure 3B, the fluorescence intensity of the DiD-DDN group on the HUVECs was significantly higher than that of the PBS group (*p* = 0.0073), further confirming the cellular uptake of DDNs by HUVECs.

### 2.4. Effects of Salvia miltiorrhiza-Derived Exosome-like Nanoparticles on the Proliferation and Migration of HUVECs 

**Figure 4:**
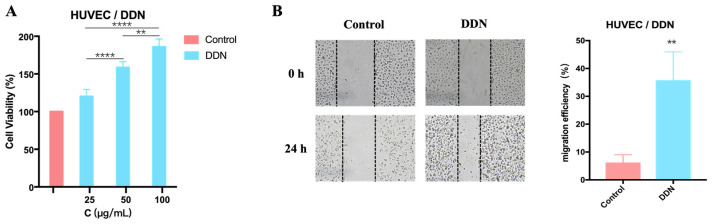
The effects of a DDN on the proliferation and migration of HUVECs; (**A**) effects of DDN on the proliferation of HUVECs (*n* = 6); (**B**) effects of DDN on the migration of HUVECs (*n* = 3). (** *p* < 0.01, **** *p* < 0.0001).

Subsequently, the effects of a DDN on the proliferation and migration abilities of HUVECs were investigated to explore its role in angiogenesis. Firstly, the effect of a DDN on HUVEC proliferation was examined using the MTT assay. The results, as depicted in Figure 4A, show that the cell viability in the DDN group was higher than that in the control group, indicating that DDNs may enhance HUVEC viability and promote cell proliferation. Furthermore, the high-dose group (100 μg/mL) exhibited a significantly better effect compared to the medium-dose (50 μg/mL) (**** *p* < 0.0001) and low-dose groups (25 μg/mL) (**** *p* < 0.0001). The effects of a DDN on the migration ability of HUVECs were assessed using a scratch wound healing assay. As shown in Figure 4B, after treatment with a high-dose DDN (100 μg/mL), the migration rate of HUVECs was significantly higher than that of the control group (*p* = 0.0011). The results suggest that DDNs can enhance HUVEC viability and promote cell migration, with the optimal concentration being 100 μg/mL.

### 2.5. Effects of Salvia miltiorrhiza-Derived Exosome-like Nanoparticles on Cardiac Function and Angiogenesis in MI/R Mice

**Figure 5:**
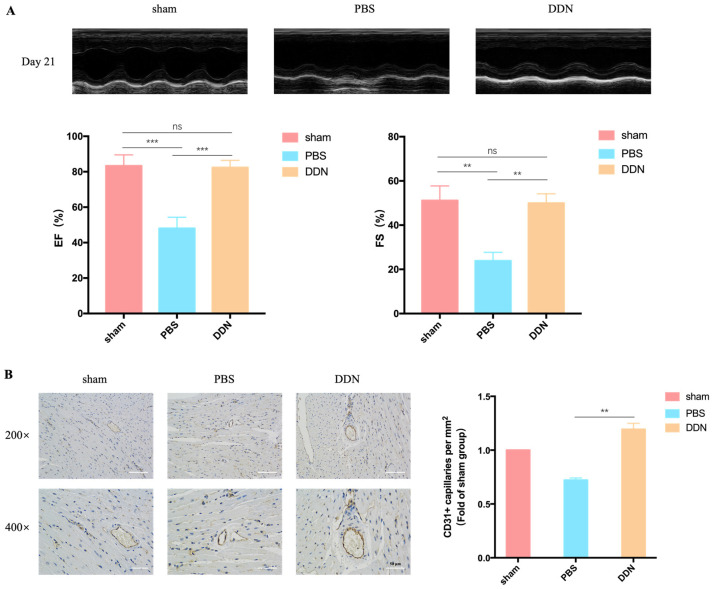
Functional analysis of DDN in vivo. (**A**) Cardiac function of C57BL/6 mice 21 days after MI/R (*n* = 3); (**B**) angiogenesis of C57BL/6 mice in each group (*n* = 3). ** *p* < 0.01, *** *p* < 0.001, ns means no significant difference, scale bar = 50 μm.

To explore the effects of DDNs on MI/R injury, the cardiac function of MI/R mice were assessed on day 21 (Figure 5A). DDNs could significantly improve the EF% and FS% compared to the PBS group 21 days post-operation, indicating that DDNs can significantly improve cardiac functions in the phase of MI/R. Immunohistochemical staining for CD31 allows for the specific visualization of endothelial cells. On postoperative day 21, cardiac tissues from each group of C57BL/6 mice were subjected to CD31 immunohistochemical staining to observe neovascularization in the peri-infarct zone of the mouse myocardium. The results, as depicted in Figure 5B, show that the microvessel density in the peri-infarct zone of the cardiac tissues of the MI/R C57BL/6 mice from the DDN group was higher compared to the PBS group (*p* = 0.0050), indicating that DDNs have a role in promoting neovascularization for MI/R treatment.

### 2.6. Safety of Salvia miltiorrhiza-Derived Exosome-like Nanoparticles

**Figure 6:**
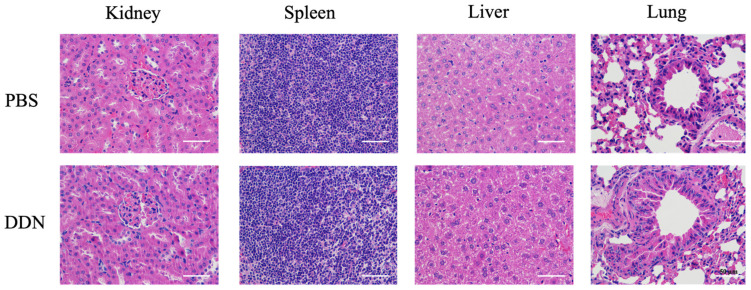
Histopathological examination of C57BL/6 mice in each group, scale bar = 50 μm.

On postoperative day 21, the kidney, spleen, liver, and lungs of the mice from each group were collected for Hematoxylin and Eosin Staining (HE Staining) to evaluate the biosafety of DDNs. A histological examination, as shown in Figure 6, revealed no significant inflammatory reactions or tissue damage in the organs of the DDN group compared to the PBS group. There were no noticeable pathological changes observed, indicating that the DDNs exhibited good safety and biocompatibility in vivo.

## 3. Materials and Methods

### 3.1. Cell Culture

HUVECs were sourced from the national collection of authenticated cell cultures (Shanghai, China). HUVECs were cultured in a Dulbecco’s-modified Eagle’s medium (DMEM) supplemented with 10% fetal bovine serum (FBS), along with 1% penicillin and streptomycin.

### 3.2. Animals

Male C57BL/6 mice (20 ± 2 g) were procured from the Slac Laboratory (Shanghai, China). All animal experiments followed the approved guidelines of the Institutional Animal Care and Use Committee at the School of Pharmacy, Fudan University (Shanghai, China).

### 3.3. Isolation and Purification of Salvia miltiorrhiza-Derived Exosome-like Nanoparticles

The isolation and purification of the DDNs were conducted using ultracentrifugation and sucrose density gradient centrifugation. Fresh *Salvia miltiorrhiza* were purchased from a *Salvia miltiorrhiza* base (Ximengshan, Weihai, China). The fresh *Salvia miltiorrhiza* were washed, adding PBS, homogenized to prepare a *Salvia miltiorrhiza* homogenate using a homogenizer, and filtered through a gauze to remove precipitates. The filtrate was centrifuged at 4 °C, 2000× *g* for 20 min. The supernatant was centrifuged at 4 °C, 12,000× *g* for 60 min, followed by an ultracentrifugation of the supernatant at 4 °C, 100,000× *g* for 90 min, and the precipitate was collected. The precipitate was resuspended in 3 mL of PBS and subjected to ultrasonication in an ice-water bath for 25 min. Subsequently, ultracentrifugation was performed again in sucrose gradients of 8%, 30%, 45%, and 60% at 200,000× *g* for 90 min. After centrifugation, the solutions from the layers of 8% and 30%, 30% and 45%, and 45% and 60% sucrose were, respectively, collected and labeled as Band1, Band2, and Band3. Then, the average particle size of Band1, Band2, and Band3 were measured using DLS with a Zetasizer nano ZS Zen3600 (Malvern, UK), and a subset with a particle size consistent with exosome dimensions was selected as the DDNs. After freeze-drying, samples were stored in a −80 °C ultra-low temperature freezer and reconstituted in PBS before the subsequent experiments. Throughout this study, the dosage of the DDN was consistently expressed as the protein concentration of the DDN, as determined by a BCA protein assay kit (Beyotime Biotechnology, Shanghai, China) when administered to animals or cells.

### 3.4. Characterization of Salvia miltiorrhiza-Derived Exosome-like Nanoparticles

An appropriate amount of a DDN freeze-dried powder was weighed and dissolved in a PBS buffer; then, the DDN was diluted with distilled water. The average particle size, PDI, and Zeta potential of the DDN were measured using DLS. Additionally, a NanoSight NS300 instrument (Malvern, Westborough, MA, USA) was employed to assess the yield of the DDN. A total of 20 μL of the DDN was placed onto a copper grid and was left to stand at room temperature for 20 min. The grid was washed three times with distilled water. Then, 20 μL of a 2% uranyl acetate solution was added, and the grid was incubated at room temperature in the dark for 20 min. The morphology of the DDN was examined by transmission electron microscopy (TEM, Tecnai G2 F20 S-Twin, FEI, Hillsboro, FL, USA). The average particle size, PDI, and Zeta potential changes of the DDN were measured and recorded on day 0, 1, 2, 3, 4, 5, 6, 7, and 30 for stability assessment.

### 3.5. Cellular Uptake of Salvia miltiorrhiza-Derived Exosome-like Nanoparticles

The DiD-DDNs were synthesized by combining 1,1′-dioctadecyl-3,3,3′,3′-tetramethyl-indodicarbocyanine perchlorate (DiD) with a DDN and incubating the mixture for 60 min at room temperature. Subsequently, a G50 Sephadex Column (GE Healthcare, Uppsala, Sweden) was employed to eliminate any unbound DiDs.

Cells were seeded onto a cell climbing slide in a 12-well plate at a density of 2 × 10^5^ cells/wells and were incubated in a cell culture incubator for 2 h until the cells adhered to the surface. Subsequently, 1 mL of a complete culture medium was added to the well, and the plate was further incubated in the cell culture incubator for 24 h. Then, the DiD-DDN was added to the well, and the plate was incubated for 4 h in the cell culture incubator under light-shielded conditions. After washing the cells twice with a pre-chilled PBS, 5 μg/mL of a Hoechst 33342 staining solution was added to the well, followed by incubation in the dark for 30 min. The cell climbing slide was observed and photographed using a laser scanning confocal fluorescence microscope (Zeiss LSM 710, Oberkochen, Germany).

To investigate the uptake of DDNs by HUVECs, the HUVECs were divided into a PBS group and a DiD-DDN group. The DiD-DDN was added to the latter group in the dark. The cell suspension was collected 4 h later, and the fluorescence intensity was measured using flow cytometry (CytoFlex S, Beckman Coulter Inc., Carlsbad, CA, USA).

### 3.6. The Effects of Salvia miltiorrhiza-Derived Exosome-like Nanoparticles on the Proliferation of HUVECs 

The HUVECs were seeded in 96-well plates at a density of 5 × 10^3^ cells per well and were cultured in a complete medium for 24 h. The HUVECs were divided into a control group, a low-dose DDN group (25 μg/mL), a medium-dose DDN group (50 μg/mL), and a high-dose DDN group (100 μg/mL). Cell viability was evaluated by MTT assay, where 100 μL of an MTT solution was applied to the cells for 4 h. Then, a DMSO solution was added and incubated for 30 min. Absorbance at 570 nm was measured using the Multiskan MK3 microplate reader (Thermo Fisher Scientific, Waltham, MA, USA).

### 3.7. The Effects of Salvia miltiorrhiza-Derived Exosome-like Nanoparticles on the Migration of HUVECs 

A 6-well plate was taken, and three horizontal lines were uniformly drawn on the back of each well using a marker pen. Subsequently, the HUVECs, in a logarithmic growth phase, were seeded into the wells of the marked 6-well plate at a density of 5 × 10^5^ cells per well and were incubated in a cell culture incubator for 24 h. Afterwards, scratches were made using a 200 μL pipette tip along the lines, with at least 3 scratches per well, and then the culture medium was replaced with a serum-free medium for continued incubation. The HUVECs were divided into a control group and a DDN group (100 μg/mL), and the scratch area was photographed using an inverted fluorescence microscope at 0 h and 24 h after administration to analyze the cell migration efficiency.

### 3.8. Establishment of MI/R Mouse Model

Following isoflurane inhalation anesthesia (1.5–2%), C57BL/6 mice underwent a longitudinal incision on the left side of the sternum between the 3rd and 4th ribs. Hemostatic forceps were employed to widen the intercostal spaces, facilitating the extrusion of the mouse heart. Ligation of the left anterior descending (LAD) artery was performed within 30 s after heart extrusion. A 7-0 silk suture was then used to ligate the LAD, which was later released after 60 min for reperfusion. The sham group underwent an identical surgical procedure, except for LAD occlusion.

### 3.9. Cardiac Protection Efficiency of Salvia miltiorrhiza-Derived Exosome-like Nanoparticles

To assess the cardiac function in the MI/R mice with DDN treatment, the postoperative mice were divided into three groups (*n* = 3): a sham group, a PBS (200 μL) group, and a DDN (10 mg/kg mouse, 200 μL) group. After 12 h of reperfusion, the mice in each group received intravenous drug injections via the tail vein for three consecutive days. Each mouse was checked with a transthoracic echocardiography under isoflurane anesthesia to analyze the left ventricular ejection fraction (EF%) and fractional shortening (FS%) on day 21 (post-MI/R).

### 3.10. Evaluation of Angiogenesis through CD31 Immunohistochemical Staining

After three weeks post-surgery, the mice were euthanized, and the left ventricle was harvested, rinsed with PBS, and fixed in a 4% paraformaldehyde solution for 24 h. The tissue was then rinsed with running water for 1 h and dehydrated using a gradient of an ethanol solution. After dehydration, the tissues were subjected to clearing by immersion in a mixed solution of xylene and anhydrous ethanol for 1 h, followed by immersion in xylene for 1 h. Subsequently, the cleared tissues were embedded in liquid paraffin and sectioned into 5–10 slices of 5 μm thickness. The sections were deparaffinized in xylene for 10 min and rehydrated using a gradient of ethanol solutions, followed by a 5 min wash in running water. The tissue sections were then immersed in an EDTA antigen retrieval buffer, washed three times with PBS, and circled with an immunohistochemistry pen. Within the circles, a 5% BSA solution was added and incubated for 30 min for blocking. After drying, the tissue sections were incubated overnight at 4 °C with a CD31 primary antibody. Following PBS washing and drying, a secondary antibody was added within the circles and incubated for 1 h. After PBS washing, the tissue sections were dried and incubated with DAB for color development, with careful monitoring under a microscope. The staining was terminated by immersion in water once the desired color was achieved. After dehydration in ethanol, clearing in xylene, and mounting with neutral resin, the tissue sections were observed under an optical microscope. Images were captured at 200× and 400× magnification, and the number of CD31-positive capillaries per unit area (mm^2^) of myocardial tissue was calculated using the ImageJ 1.5.4 software.

### 3.11. Assessment of the Safety of Salvia miltiorrhiza-Derived Exosome-like Nanoparticles

HE Staining was used to assess the safety of DDNs in MI/R mice. After three weeks post-surgery, the mice from each group were euthanized, and major organs such as the liver, spleen, kidney, and lungs were collected for paraffin section preparation. After deparaffinization and hydration, the sections were stained with hematoxylin for 15 min, followed by a 5 min rinse in running water. Differentiation was performed using 1% hydrochloric acid alcohol, followed by bluing in Scott’s tap water solution. Eosin staining was performed for 2 min, followed by a 5 min rinse in running water. Dehydration was carried out using 95% and 100% ethanol solutions, followed by clearing in xylene and mounting with neutral resin. The tissue sections were observed under an optical microscope, and images were captured at 200× and 400× magnification.

### 3.12. Statistical Analysis

Significant differences were evaluated using an unpaired Student’s *t*-test for a comparison of two groups and one-way ANOVA for multiple-group comparisons. The data are expressed as a mean ± standard deviation (SD), and a *p* value of < 0.05 was considered to indicate a significant difference (* *p* < 0.05, ** *p* < 0.01, *** *p* < 0.001, and **** *p* < 0.0001).

## 4. Discussion

We discovered exosome-like nanoparticles (DDNs) in *Salvia miltiorrhiza*, which have been proven to promote angiogenesis and exhibit therapeutic effects in MI/R injury.

Establishing stable methods for exosome extraction and characterization is a prerequisite for exosome research. The isolation and purification of exosomes are often based on their density characteristics, using methods such as ultracentrifugation or sucrose density gradient centrifugation to separate and extract exosomes [38]. Ultracentrifugation is simple, time-saving, and energy efficient, with a high extraction efficiency, making it suitable for extracting exosomes from mammalian cells with low contents [39]. Sucrose density gradient centrifugation is a zone-separation method, where exosomes mainly concentrate in a density band of 1.13–1.20 g/mL. Although this method yields lower extraction volumes, the purified exosomes have high purity and a more uniform particle size distribution. However, sucrose density gradient centrifugation requires strict centrifugation conditions and involves complex steps, limiting its application [36,40]. In this study, we improved upon existing methods by first using ultracentrifugation to achieve the large-scale enrichment of exosome-like nanoparticles from *Salvia miltiorrhiza*, followed by a further separation and purification using sucrose density gradient centrifugation. Firstly, the choice of sucrose concentrations was based on previous literature reports that have demonstrated a successful purification of plant-derived exosome-like nanoparticles using 8%, 30%, 45%, and 60% sucrose gradients [28,29]. Based on the density characteristics of exosomes in the range of 1.13–1.20 mg/L, DDNs were obtained between the layer of an 8% and 30% sucrose solution. This method ensures the large-scale extraction of DDNs while maintaining a uniform particle size distribution and high purity. Extracting exosomes from mammalian cells typically requires 4–5 days of a cell culture to produce sufficient exosomes for extraction. In contrast, extracting exosome-like nanoparticles from *Salvia miltiorrhiza* can be completed within one day, significantly improving extraction efficiency. Additionally, every 1 kg of *Salvia miltiorrhiza* can yield 961 mg of exosome-like nanoparticles (based on protein content), demonstrating high extraction yields. The characterization of exosomes involves a relatively complete process, including observing their morphological characteristics using TEM, measuring concentrations using NTA, and analyzing particle sizes and Zeta potentials using DLS. We characterized the purified DDN using DLS, revealing an average particle size of 105.15 nm with a uniform particle size distribution. The Zeta potential of the DDN was −25.2 mV, indicating a negative charge similar to the cell membrane. The TEM analysis showed that DDNs have a predominantly spherical shape with a membrane-enclosed vesicle-like structure. After storage at −80 °C for one month, the particle size of the DDN fluctuated between 100 nm and 140 nm, and the Zeta potential fluctuated between −20 mV and −25 mV, demonstrating a certain stability. Similar to our research findings, the average diameter of *Panax notoginseng*-derived exosome-like nanoparticles extracted using sucrose density gradient centrifugation was approximately 151.3 nm [29], while that of ginseng-derived exosome-like nanoparticles had an average diameter of approximately 151.6 nm, with a potential of −17.9 mV [28]. This indicates that the DDN extracted in our study possess physicochemical properties similar to other plant-derived exosome-like nanoparticles, laying a foundation for future research.

Plant-derived exosome-like nanoparticles have been proven to be safe biological agents with therapeutic effects on various diseases [25,26]. It is worth noting that exosome-like nanoparticles have been widely extracted from various edible plants, such as ginger, *Panax notoginseng*, and ginseng. These nanotherapies have been demonstrated to have therapeutic effects on diseases such as colitis, strokes, and cancer, which are similar to the biological effects of ginger, *Panax notoginseng*, and ginseng [27,28,29]. It is recommended and reliable to select the source of plant-derived exosome-like nanoparticles based on the original biological activity of plants. Therefore, we speculate that a DDN also has a similar promoting effect of *Salvia miltiorrhiza* on angiogenesis, thereby reducing MI/R injury. However, there is currently no research on extracting exosome-like nanoparticles from *Salvia miltiorrhiza*, nor is there any evidence supporting the therapeutic effects of plant-derived exosome-like nanoparticles on MI/R injury. To investigate the role of DDNs in MI/R injury, we used HUVECs to study the effects of DDNs on promoting angiogenesis in vitro. The choice of HUVECs for cellular uptake, proliferation, and migration studies is based on their relevance to angiogenesis and physiological similarity to endothelial cells in vivo [41]. We confirmed that DDNs can promote the proliferation and migration of HUVECs in vitro and increase microvessel density in MI/R mice. As an angiogenesis promoter, DDNs have a certain efficacy in the treatment of MI/R injury. This lays the foundation for future research on the role and mechanism of DDNs in the treatment of MI/R injury.

We have successfully extracted exosome-like nanoparticles from *Salvia miltiorrhiza*, elucidating the pharmacology of DDNs against MI/R injury. This not only lays an experimental foundation for the development of *Salvia miltiorrhiza* but also expands the applicational scope of plant-derived exosome-like nanoparticles. However, this study is not comprehensive, lacking detailed in vivo research to thoroughly assess the efficacy and biodistribution of DDNs. In the future, an exploration of the potential of the oral administration of DDNs for treating MI/R injury could be considered to further maximize the applicational value of DDNs.

## 5. Conclusions

This study established a comprehensive method for the extraction and characterization of *Salvia miltiorrhiza*-derived exosome-like nanoparticles (DDNs). DDNs were successfully extracted from *Salvia miltiorrhiza* using ultracentrifugation and sucrose density gradient centrifugation methods. Morphological characteristics of the DDNs were observed using TEM, while their particle size distribution, Zeta potential, and concentration were analyzed using DLS and NTA. The average particle size of the DDNs was 105.15 nm, with a uniform size distribution. The Zeta potential of the DDNs was −25.2 mV, and the DDNs had a predominantly spherical shape with a membrane-enclosed vesicle-like structure. Meanwhile, we confirmed that DDNs can promote the proliferation and migration of HUVECs in vitro, as well as increase the microvascular density of MI/R mice in vivo, thereby alleviating MI/R injury. Additionally, DDNs demonstrate a certain level of safety in vivo. The DDNs extracted using this method exhibited stable properties of exosome-like nanoparticles and demonstrated angiogenic effects, laying the foundation for their potential application in the treatment of MI/R injury.

## Data Availability

Data are contained within the article.
